# Pollinator‐mediated selection on floral size and tube color in *Linum pubescens*: Can differential behavior and preference in different times of the day maintain dimorphism?

**DOI:** 10.1002/ece3.3683

**Published:** 2017-12-20

**Authors:** Merav Lebel, Uri Obolski, Lilach Hadany, Yuval Sapir

**Affiliations:** ^1^ The Botanical Garden Tel Aviv University Tel Aviv Israel; ^2^ School of Plant Sciences and Food Security Tel Aviv University Tel Aviv Israel; ^3^ Present address: Science Division Nature‐Parks Authority Jerusalem Israel; ^4^ Present address: Department of Zoology University of Oxford Oxford UK

**Keywords:** Bombyliidae, divergent selection, insect behavior, maternal fitness, mating rendezvous, phenotypic variation, pollinator‐mediated selection

## Abstract

Diversity of flower traits is often proposed as the outcome of selection exerted by pollinators. Positive directional pollinator‐mediated selection on floral size has been widely shown to reduce phenotypic variance. However, the underlying mechanism of maintaining within‐population floral color polymorphism is poorly understood. Divergent selection, mediated by different pollinators or by both mutualists and antagonists, may create and maintain such polymorphism, but it has rarely been shown to result from differential behavior of one pollinator. We tested whether different behaviors of the same pollinators in morning and evening are associated with dimorphic floral trait in *Linum pubescens,* a Mediterranean annual plant that exhibits variable within‐population frequencies of dark‐ and light‐colored flower tubes. *Usia bicolor* bee‐flies, the major pollinators of *L. pubescens*, are mostly feeding in the flower in the morning, while in the evening they are mostly visiting the flowers for mating. In 2 years of studying *L. pubescens* in a single large population in the Carmel, Israel, we found in one year that dark‐centered flowers received significantly higher fraction of visits in the morning. Fitness was positively affected by number of visits, but no fitness differences were found between tube‐color morphs, suggesting that both morphs have similar pollination success. Using mediation analysis, we found that flower size was under positive directional pollinator‐mediated selection in both years, but pollinator behavior did not explain entirely this selection, which was possibly mediated also by other agents, such as florivores or a‐biotic stresses. While most pollinator‐mediated selection studies show that flower size signals food reward, in *L. pubescens*, it may also signal for mating place, which may drive positive selection. While flower size found to be under pollinator‐mediated selection in *L. pubescens*, differential behavior of the pollinators in morning and evening did not seem to explain flower color polymorphism.

## 1 INTRODUCTION

Flowers of animal‐pollinated plants show great diversity in shape and color among families, genera, and species and sometimes even among and within populations of the same species (Narbona, Wang, Ortiz, Arista, & Imbert, 2017). This wide floral variation is considered to be the result of their interactions with pollinators (Darwin, 1862; Fægri & van der Pijl, 1979). Flowers signal to pollinators by a variety of cues and the extent of fertilization success associated with the variance of a trait creates selection on this trait (Aigner, 2006; Harder & Johnson, 2009; Schiestl & Johnson, 2013; Sletvold, Grindeland, & Ågren, 2010). Much attention was given in the last decades to the quantification of the extent of selection exerted by pollinators on various floral traits, including flower size, color, and scent (Harder & Johnson, 2009; Schiestl & Johnson, 2013). However, the mechanisms maintaining within‐population variation are much less understood and are not necessarily pollinator‐mediated (Gigord, Macnair, & Smithson, 2001; Imbert et al., 2014; de Jager & Ellis, 2014; Lau & Galloway, 2004; Melendez‐Ackerman, Campbell, & Waser, 1997; Rymer, Johnson, & Savolainen, 2010).

Floral size acts as an important visual signal for the pollinators, enabling the detection by pollinators from a substantial distance (e.g., Eckhart, 1991; Hegland & Totland, 2005; Mitchell, Karron, Holmquist, & Bell, 2004). Thus, pollinators can exert positive selection on floral size (Conner & Rush, 1996; Lavi & Sapir, 2015; Stout, 2000). On the other hand, negative pollinator‐mediated selection on floral size can be manifested through handling time, because large flowers can make it harder for a pollinator to find its way to the reward, which may lead to a preference for smaller flowers (Sutherland, Sullivan, & Poppy, 1999). In addition, in specific plant‐pollinator interactions, stabilizing selection may reduce floral variance to ensure the best fit between pollinator and flower (van Kleunen, Meier, Saxenhofer, & Fischer, 2008). While pollinators usually tend to visit plants with larger advertisement (either flowers or inflorescences), improving handling of the flowers requires other floral features. In zygomorphic flowers, it is hypothesized that bilateral symmetry enhances pollinators’ learning (Giurfa, Dafni, & Neal, 1999; Neal, Dafni, & Giurfa, 1998), while in radial flowers, the orientation of the pollinator within the flower can be improved by color patterns of the flower (Johnson & Dafni, 1998).

A dark flower center was proposed to play a role in attracting pollinators as an orientation mark toward the location of both reproductive organs and reward (e.g., Dafni et al., 1990; Ellis & Johnson, 2010; Ellis et al., 2014; de Jager & Ellis, 2012, 2014; Johnson & Dafni, 1998; Johnson & Midgley, 1997, 2001; Keasar et al., 2010; Papiorek et al., 2016). Darwin (1888) proposed that a dark flower center is of no functional importance to the plant and it may reflect past adaptation that is not functioning in present environment. Despite Darwin's view, several hypotheses have been proposed to explain the role of dark flower center in the context of pollination. For instance, it was hypothesized that the dark center imitates an insect to attract other insects (Eisikowitch, 1980; Ellis & Johnson, 2010; de Jager & Ellis, 2012; Johnson & Midgley, 1997; Lamborn & Ollerton, 2000). Nonetheless, studies in Apiaceae species with a dark spot of sterile florets in the center of the inflorescence showed contrasting results. For example, houseflies (*Musca domestica*) were more attracted to inflorescences of *Daucus carota* with a dark spot, and adding dead flies to inflorescences without dark spot increased number of visits of flies (Eisikowitch, 1980). In another study, however, Sawflies (*Tenthredo* sp.) attacked the dark central florets of *D. carota*, which suggests that the dark spot may attract predator insects (Lamborn & Ollerton, 2000). Johnson and Midgley (1997) found that flowers of *Gorteria diffusa* have spots on the ray florets, which are strikingly similar to its pollinator, a small bee‐fly, *Megapalpus nitidus* (Bombyliidae). These flies are attracted to the spot and try to copulate with it, leading to the conclusion that the dark spots imitate flies to attract males looking for mating (Johnson & Midgley, 1997). The wide distribution of plants with a dark‐center pattern and the recorded behavior of pollinators attracted to these patterns suggest that dark flower center has an adaptive role in pollination.

We propose that the dark center in fly‐pollinated flowers is important to attract and to guide pollinators and that this trait facilitates pollinator‐mediated selection on floral size. Guiding the pollinator may reduce handling time and facilitate directional selection on size. This leads to the hypothesis that dark‐center flowers will experience stronger positive directional selection on size, compared to flowers without dark center. In order to test the role of both dark flower center and flower size, we studied pollinator‐mediated selection on floral tube color and size in a Mediterranean annual plant. Specifically, we addressed the following questions: (1) Does variation in floral size and tube color traits affect visitation rates? (2) Does variation in floral traits affect pollinator's behavior? (3) Does fitness correlate with fly visitation rates? (4) Is there pollinator‐mediated selection on floral size and tube color? We hypothesize that floral size is under positive directional selection, mediated by the pollinators, while selection on floral tube color is expected to be dependent on pollinators’ different behaviors, associated with specific color preference.

## 2 MATERIALS AND METHODS

### 2.1 Study system

**Figure 1:**
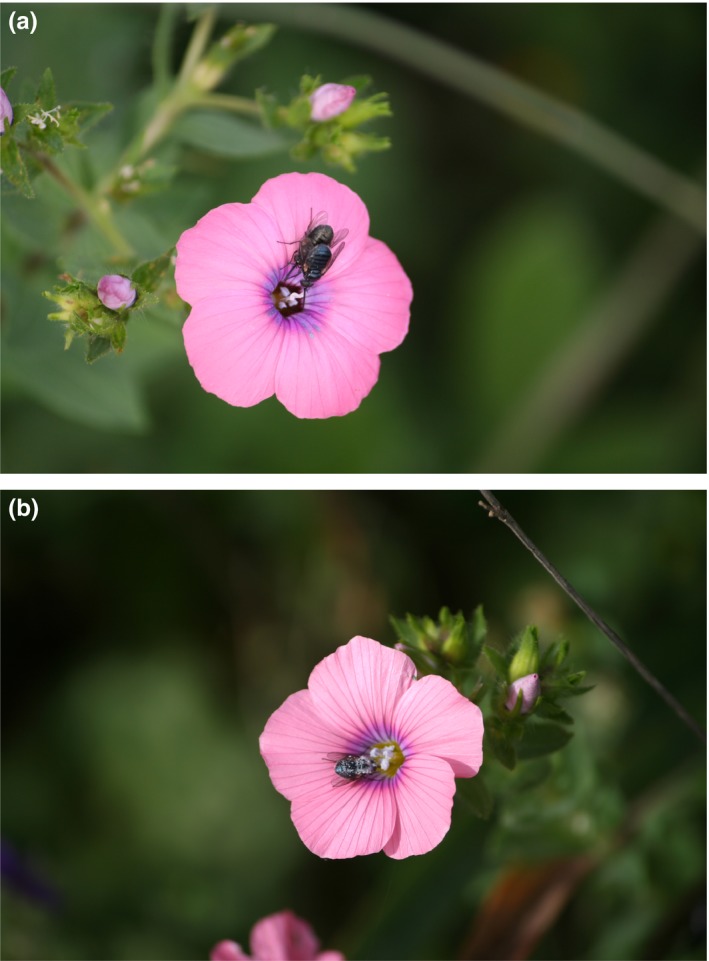
Flowers of *Linum pubescens* with the major pollinator, *Usia bicolor* bee‐fly. (a) Flower with dark tube with two flies mating; (b) Flower with light‐colored tube with a feeding fly

*Linum pubescens* Banks & Sol. (Linaceae) is an annual herbaceous plant growing in the Eastern Mediterranean region and is highly abundant in the Mediterranean climatic region in Israel. The plant has a few flowers open daily, each lasting between 2–4 days (Dulberger, 1973; M. Lebel, unpublished data). Flowers of *L*. *pubescen*s are dimorphic in the color of the petal bases, forming either dark‐ or light‐colored tube (‎Figure 1). The ratio between color morphs is variable among populations, and usually, light‐colored flowers dominate in populations (Wolfe, 2001).

The main pollinator of *L. pubescens* is the bee‐fly *Usia bicolor* (Diptera: Bombyliidae; Johnson & Dafni, 1998). Flies forage mostly for nectar during the morning, and in the afternoon, they visit the flowers mostly for mating (Johnson & Dafni, 1998). A laboratory experiment showed that *U. bicolor* flies visited flower models with a dark spot only during the evening and that they preferred larger flower models (Johnson & Dafni, 1998). Based on these results, we expected that flies will prefer dark‐centered flowers when searching for mating and light‐centered flowers (or none) when searching for food. This predicts a mixed selection regime on floral color in *L. pubescens*.

### 2.2 Research site

The research was performed during peak flowering time of *L. pubescens*, in the spring of 2010 (February 15^th^ to March 25^th^) and 2011 (March 20^th^ to April 18^th^). Due to high interannual variation in the climate in the semi‐dry Mediterranean climate region, peak flowering was about 3 weeks later in 2011, compared to 2010. The study was performed in Ramat Hanadiv Nature Park, located in the Southern part of the Carmel mountain ridge (32º33′14′’N, 34º56′38′’E; altitude 125 meters above sea level). The climate there is Mediterranean with an average annual precipitation of 541 mm. Average temperatures range between 8.7°C and 17.5°C in February, 10.5°C and 19.8°C in March, and 15.8°C and 24°C in April. Vegetation is mainly Mediterranean woodland dominated by *Pistacia lentiscus, Calicotome villosa,* and *Sarcopoterium spinosum*. The population of *L. pubescens* is relatively large and dense in this site. Effective population size of *L. pubescens* is almost impossible to estimate because as an annual herb it is spread over all natural areas in the Mediterranean region in Israel, with different patch densities that vary among years, probably due to seed dormancy (M. Lebel, unpublished data). The sampling was performed in an area of about 0.5 km^2^, where *L. pubescens* is abundant.

### 2.3 Floral traits

We sampled flowers along four 30‐meter long transects, chosen by randomly assigned directions and starting points. Each year, plants along these transects were marked individually by numbers written on paper tags. Note that these plants are annual, hence, different plants were assigned to the study each year. Moreover, seeds of *L. pubescens* exhibit strong dormancy, where only ~2% of the seeds germinate in the next season (M. Lebel, unpublished data); hence, we assumed that the sampled plants in the different years were independent. The number of open flowers per plant ranged between 1 and 8, and this number was different among plants and days. One open flower was chosen randomly in each plant and its diameter was measured with a digital caliper (accuracy 0.05 mm). Flower diameter was remeasured, each day observations were made, and the variation within each plant was negligible (data not shown). Tube color was similarly assessed for each plant at every observation and showed no within‐plant variation.

### 2.4 Insect visitation

Flowers of *L. pubescens* are closed during the night and open 2–3 hours after sunrise. Pollinators’ activity on flowers of *L. pubescens* is restricted to morning and late afternoon (Johnson & Dafni, 1998; Lebel, 2011), confirmed also in observations during this study. Hence, observations were performed on all marked plants twice a day, in the morning, after the flowers opened (usually 9:00–11:00 a.m.), and about one hour before flowers started to close (usually around 3:30–5:30 p.m.). A pollinator's visit was determined when an insect touched any part of the petals in a flower of a marked individual plant.

In each observation session, an observer walked along the predefined transects and recorded all the insects that visited each of the individually marked plants. Both number and identity (to the level of order) of the insects in the flowers were recorded. Because sampling was performed during walking along transects, the duration of visits was not recorded.

In a preliminary study, we observed flowers for intervals of 60 min along the day, in order to determine peak period of activity and to determine the major behaviors. Following these observations, insect behavior was categorized as follows: (1) Feeding—insect probing in and out the flower's tube. (2) Mating—at least one insect stays within the tube for a long time (more than 5 minutes; assessed in the preliminary observations), where almost in all cases another insect joins and copulation occurs. (3) Waiting—insect stays on the petals (outside the tube), without entering the tube. (4) Other—undefined behavior, such as brief touch of the petals or standing on the petals’ back side.

Samples of visiting insects were collected from flowers and were identified by Dr. Amnnon Freidberg and Mr. Leonid Friedman at the Steinhardt Museum of Natural History, Tel Aviv University and by Mr. David Gibbs (http://davidjgibbs.webs.com/). All flies collected in these observations were identified as *U. bicolor*. Assessment of insects’ gender was not performed in the field, because body size and other visible characteristics are not associated with sex in this species (D. Gibbs, personal communication).

Mean number of visits per plant was calculated as the number of observations where the flowers of that plant were hosting at least one insect, divided by the number of observations of that plant, with or without insects.

### 2.5 Fitness

In order to measure plants’ female fitness, all marked plants were collected at the end of the season (mid‐May) in both years. In a previous study, on a subsample of these plants (Bigio, Lebel, & Sapir, 2016), we found that the number of fruits and the number of seeds are highly correlated (Pearson's *r* = 0.96). Hence, we used the number of fruits per plant in this study as a measure of maternal fitness. In order to estimate Lande and Arnold's (1983) natural selection, we calculated the relative fitness of a plant as the number of fruits from that plant divided by the overall mean number of fruits in the population sample, separately for each year. Male fitness, such as pollen uptake or seeds sired, was not evaluated in this study.

### 2.6 Statistical analysis

In order to test whether pollinators differentially prefer any of the color morphs in the morning or in the evening, while controlling for their relative proportions, we used differences in ratios (DR) of visits. Because observations of visits in the morning and evening were made to the same population of plants, and putatively by the same visitors, there is potentially high interdependency between the data points. In addition, the proportions of dark‐ and light‐color tubes were not equal. Thus, we used this DR to compare directly the preferences of pollinators to tube color in the different times of the day, based on the proportions of visits. To compare the ratios, we first calculated the ratio between visits to dark‐tube plants and total visits, separately for morning and evening time. Next, we subtracted the ratio for the morning from the ratio for the evening, to calculate the differences of ratios (DR):

(1) $$\begin{array}{cc} \text{DR}= & \left[\frac{\text{x(dark,}\;\text{evening)}}{\text{x(dark,}\;\text{evening)}\;+\;\text{x(light,}\;\text{evening)}}\right]-\\ & \;\left[\frac{\text{x(dark,}\;\text{morning)}}{\text{x(dark,}\;\text{morning)}\;+\;\text{x(light,}\;\text{morning)}}\right] \end{array}$$

where *x* is the mean number of visits per plant. DR values of zero indicate no preference to dark‐tube color at any time of the day, while positive values indicate preference of pollinators to dark‐tube color in the evening, and negative values indicate preference for dark‐tube color in the morning. We used bootstrap resampling (*boot* function in R) to estimate the distribution of the DR statistic and assess whether it is statistically different from zero.

In order to test for the response of pollinators to floral traits in different times of the day, we used an analysis of covariance, with mean number of visits (averaged per plant, across all observation days) as the response variable and floral diameter, tube color, and their interactions with time of the day as the explanatory variables. Next, we tested for different selection regimes on flower tube color and size in the morning and the evening. We used analysis of covariance with relative fitness as the explanatory variable, and morning and evening visitations, as well as floral traits and their interactions with either morning and evening visitation as the explanatory variables. In both of the above tests, we used generalized linear models (GLMs) and verified the results with sampling randomization (bootstrap) to assess significance, because of non‐normal distribution of the models’ residuals (see Results).

To assess the role of pollinators as mediators of selection between a floral trait and a fitness measure, we used the framework of mediation analysis (Gong & Huang, 2009). Mediation analysis aims to identify the role of intermediate factors (mediators) that are affected by an explanatory variable and are affecting the response variable (Baron & Kenny, 1986). A mediator is defined as a variable that is hypothesized to explain the relationship between a predictor and an outcome. This method is preferable over path analysis, which also uses intermediate variables, when the explanatory variables are categorical and not continuous (Mitchell, 2001).

To assess the role of pollinators as mediators between floral traits and fitness, we performed mediational analysis in four steps, using three linear models (Gong & Huang, 2009):

Step 1: fitness as a function of floral trait
Step 2: pollinator behavior as a function of floral trait
Step 3: fitness as a function of pollinator behavior
Step 4: fitness as a function of floral trait and pollinator behavior

The first two steps were implemented separately using either one‐way analysis of variance (ANOVA) for color or simple linear regression for flower diameter. The last two steps were implemented together using a two‐way ANOVA or multiple regression for color or flower diameter, respectively.

All statistical analyses were performed in R (R Development Core Team 2014).

## 3 RESULTS

Of 298 *L. pubescens* plants measured in 2010, 32% had dark‐tube flowers. A similar proportion was found in 2011, where 33% of 251 plants measured were dark‐tube flowers. Average floral size was 22.9 mm in 2010 (range from 12.0 to 31.0 mm) and 26.4 mm in 2011 (range from 15.4 to 34.1 mm). In both years, there was no significant difference in flower size between dark‐ and light‐color flowers (*t* test: *p* > .4).

As mentioned above, individually marked plants were observed twice a day for a few days. Total of 1,297 observations on 298 plants was made in 2010 (note that many plants were observed multiple times in different days); a visit was recorded in 24.9% of the observations. A larger proportion of the plants were visited in the morning, while of the total visitation percentage, there were more visitations in light‐centered flowers (Table 1). In 2011, 2,339 observations were made on 251 plants, and a visit was recorded in 23% of these. Similar to 2010, a higher fraction of individuals were visited in the morning. The total visitation percentage was slightly higher in dark‐tube flowers (Table 1).

**Table 1:** Number of individual *Linum pubescens* plants of two color morphs visited by *Usia bicolor* in different times of the day in two research years (2010 and 2011)

Year	Time at day	Dark	Light	Percentage of observations with visit (%)
2010 (n = 298)	Morning	39	166	30.5
	Evening	26	92	18.8
	Percentage per color morph	65 (19.2%)	258 (26.9%)	24.9
2011 (n = 251)	Morning	93	170	25.8
	Evening	95	180	20.8
	Percentage per color morph	188 (23.7%)	350 (22.7%)	23

n—Number of individual plants observed. Percentages denote fraction of observations with visit out of all. Note that plants were observed multiple times (see text).

In 2010, we recorded 401 insects visiting *L. pubescens* flowers, of which 380 (95%) were *U. bicolor*, while the rest were six beetles (mainly from the family Meloidae, subfamily Cetoniinae), 13 grasshoppers (mainly the genus *Isophya* from the family Tettigoniidae), one unidentified solitary bee, and one unidentified bee‐fly. In 2011, we recorded 869 insects visiting *L. pubescens* flowers, of which 845 (97%) were *U. bicolor*, and the rest (3%) consisted of two beetles, 21 solitary bees, and one unidentified bee‐fly. Because *U. bicolor* was the main visitor in *L*. *pubescens* flowers, further analyses were performed using only *U. bicolor*.

Similar to the observations of Johnson and Dafni (1998), *U. bicolor* flies exhibited different behaviors in different times of the day when visiting in *L. pubescens* flowers. In 2010, 21% of the visitors in the morning were feeding in the flowers, 37% were mating, another 37% were recorded as waiting, and 5% performed other behaviors. In cold mornings, when the air temperature was <18°C, many flowers contained two or more flies. This morning mating‐like behavior was observed in cold mornings only, whereas additional observations confirmed that flies that mated in the previous evening stay in the flower through the next morning in low temperatures. When air temperature in the morning exceeded 18°C, night staying flies emerged earlier. In further analyses, records of mating‐like behavior in cold mornings (<18°C) were omitted from the morning data. In the evening, most of the visitors (86% of visits) came to the flowers for mating, and no feeding behavior was observed.

In 2011, most morning visitors were feeding (50%) or waiting in the flowers (44.5%), with only 4.5% of the visitors performing mating behavior and 1% of the visitors performing other behaviors. In contrast, only 2% of the visitors in the evening were feeding, while 72% were mating in the flowers, and 22% were waiting. Following these differences between morning behavior (feeding) and evening behavior (mating), we hereafter refer to morning visitations and evening visitations as a proxy for the two behaviors.

### 3.1 Tube color and size effects on visits

**Figure 2:**
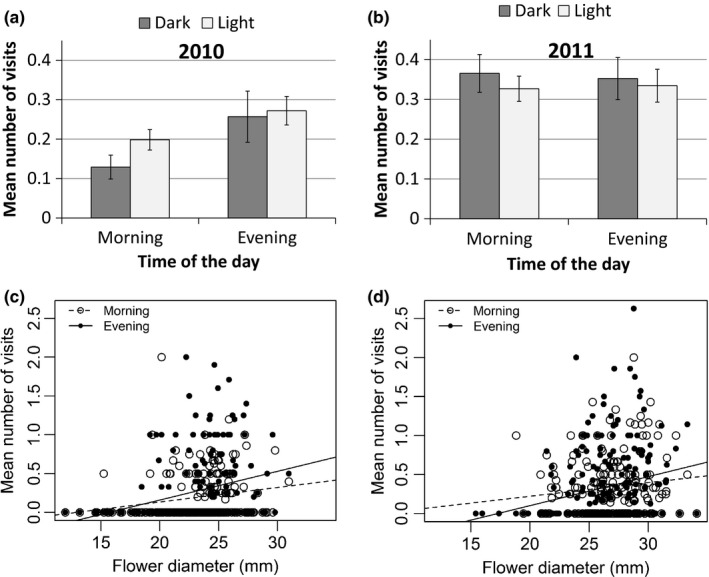
Differences between morning and evening in number of *Usia bicolor* flies visits in flowers of *Linum pubescens* as a function of floral traits. (a) Mean number of visits as a function of tube color in 2010. Bars are means ± standard errors. (b) Mean number of visits as a function of tube color in 2011. Bars are means ± standard errors. (c) Mean number of visits per plant as a function of flower diameter in 2010. (d) Mean number of visits per plant as a function of flower diameter in 2011

Analysis of difference of ratio (DR) of visits to dark‐tube flowers in the morning and evening revealed no significant difference in 2010 (DR=0.143, *p* = 0.169; Figure 2a). In contrast, in 2011, we found significant deviation of the ratios from zero (DR= −0.184, *p* = .04; Figure 2b). This implies higher preference for dark‐centered flowers in the morning.

Although flower diameter significantly affected visitation rates in both years (Table 2), the effect of flower diameter on the number of visits was not significantly different between the morning and evening visitations (Analysis of covariance: *p* = .24 for 2010 and *p* = .37 for 2011; Table 2). The number of visits per plant was not significantly affected by tube color and was not different between morning and evening (Table 2).

**Table 2:** Analysis of covariance table for number of visits per plant as a function of flower diameter and color and the time of the day (morning and evening)

Year	Source	*df*	SS	*F*	Sig. *p*
2010	Time	1	0.62	4.51	.034 (.13)
	**Flower diameter**	1	3.28	23.95	<.001 (<.001)
	Tube color	1	0.21	1.57	.211 (.38)
	Flower diameter x tube color	1	0	0.01	.910 (.77)
	Time x Flower diameter	1	0.35	2.54	.112 (.24)
	Time x Tube color	1	0	0.01	.939 (.72)
	Residuals	382	52.2		
2011	Time	1	0	0	.998 (.67)
	**Flower diameter**	1	2.70	14.63	<.001 (.001)
	Tube color	1	0.11	0.61	.435 (.55)
	Flower diameter x tube color	1	0.31	1.67	.196 (.44)
	Time x Flower diameter	1	0.31	1.66	.196 (.37)
	Time x Tube color	1	0	0.01	.909 (.69)
	Residuals	391	72.03		

*p* ‐values from a linear model are accompanied by *p* ‐values that were obtained using bootstrap (in parentheses), to account for the non‐normal distribution of the data. Significant effects are shown in bold.

Analysis of covariance revealed a significant effect of flower diameter on the relative fitness in both years (Table 3), but no statistically significant effect was observed for tube color (Table 3). In 2010, morning visitations significantly affected fitness, but no interaction was found between visitations and either flower diameter or flower tube color (Table 3). In 2011, both morning and evening visitations significantly affected fitness. In both years, interactions between floral traits and either morning or evening visitations were not significant in their effect on fitness (Table 3).

**Table 3:** Analysis of covariance table for fitness as a function of flower diameter, tube color, and visits in different times of the day (morning and evening)

Year	Source	*df*	SS	*F*	Sig. *p*
2010	**Flower diameter**	1	14.5	18.90	<.001 (<.001)
	Tube color	1	0.7	0.97	.326 (.469)
	**Morning visits**	1	10.3	13.44	<.001 (.006)
	Evening visits	1	5.2	6.72	.010 (.137)
	Flower diameter x morning visits	1	1.5	1.91	.169 (.388)
	Flower diameter x evening visits	1	0.04	0.82	.819 (.582)
	Tube color x morning visits	1	1.8	2.35	.127 (.379)
	Tube color x evening visits	1	0.1	0.17	.679 (.512)
	Residuals	164	125.6		
2011	**Flower diameter**	1	34.6	51.7	<.001 (<.001)
	Tube color	1	2.9	4.35	.039 (.188)
	**Morning visits**	1	36.3	54.27	<.001 (<.001)
	**Evening visits**	1	24.5	36.58	<.001 (<.001)
	Flower diameter x morning visits	1	2.4	3.53	.061 (.182)
	Flower diameter x evening visits	1	2.5	3.71	.056 (.200)
	Tube color x morning visits	1	4.7	7.10	.008 (.129)
	Tube color x evening visits	1	1.3	1.95	.164 (.386)
	Residuals	175	116.9		

*p* ‐values from a linear model are accompanied by *p* ‐values that were obtained using bootstrap (in parentheses), to account for non‐normal distribution of the data. Significant effects are shown in bold.

### 3.2 Pollinator‐mediated selection on flower tube color and size

**Figure 3:**
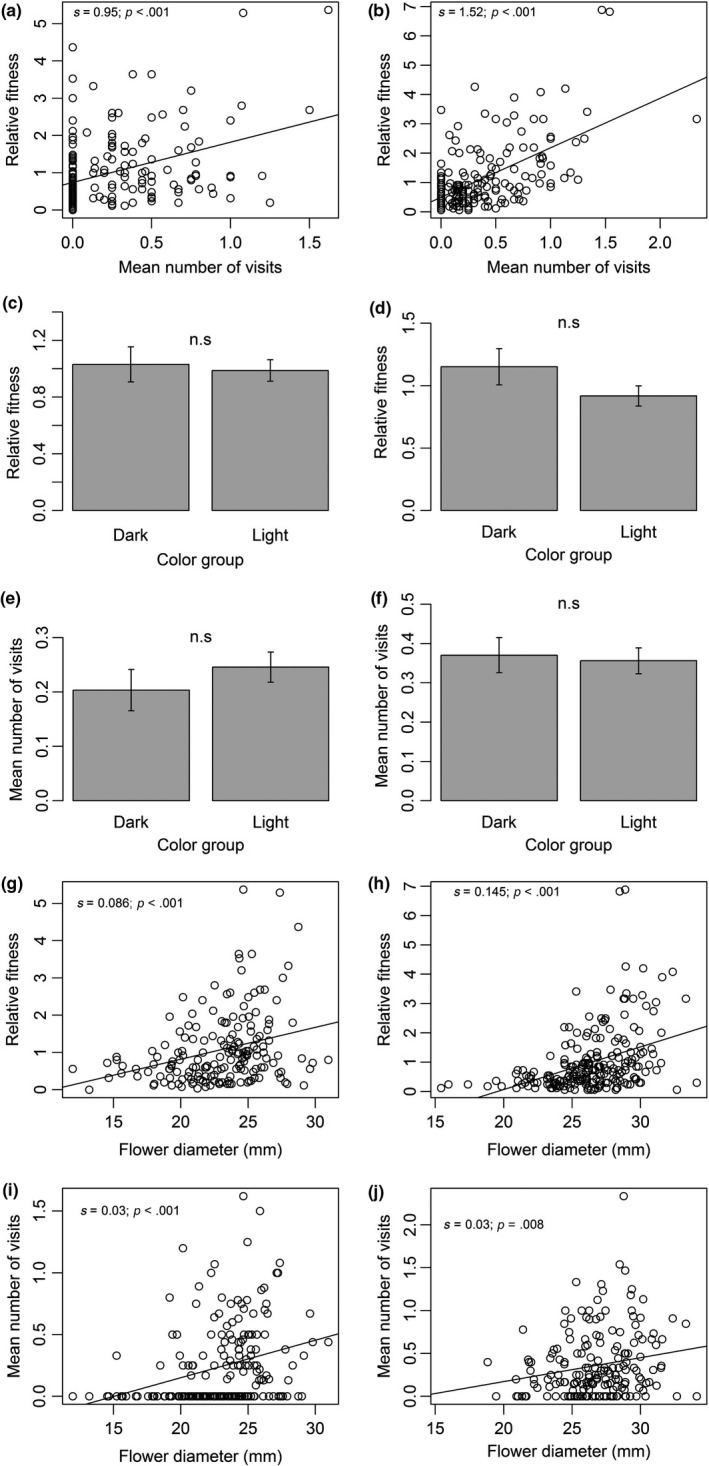
Associations between floral traits (tube color and flower diameter), pollinators behavior (mean number of visits per flower), and relative fitness (number of fruits) in *Linum pubescens* in 2010 (left) and 2011 (right). n.s.—nonsignificant difference (*p* > .05); s—slope of the regression

Mediation analysis for pollinator‐mediated selection on flower tube color and size was similar between years (Table 4). In both years, fitness was positively affected by the mean number of visits (Figure 3a,b). However, color morphs did not differ in mean number of visits per plant, as well as in mean relative fitness, in both years (Figure 3c–f). This implies that pollinators are not the mediators of selection, if it exists on tube color. Flower diameter, on the other hand, was found to be under positive pollinator‐mediated selection in both years. Flower diameter affected both fitness and pollinator behavior (Figure 3 g–j).

**Table 4:** Analyses to test the mediator effect of pollinator behavior on the association between floral traits and fitness in *Linum pubescens*

Predictor variable	Explained variable	Effect size	Sig. *p*
2010			
Color→visits→fitness			
Step 1: Color	Relative fitness	B = −0.04	.996
Step 2: Color	Mean # of visits	B = 0.04	.403
Step 3: Mean # of visits	Relative fitness	s = 1.09	**<.001**
Color	Relative fitness	s = −0.09	.739
Flower diameter→visits→fitness			
Step 1: Flower diameter	Relative fitness	s = 0.09	**<.001**
Step 2: Flower diameter	Mean # of visits	s = 0.03	**<.001**
Step 3: Mean # of visits	Relative fitness	s = 0.95	**<.001**
Flower diameter	Relative fitness	s = 0.06	**<.041**
2011			
Color→visits→fitness			
Step 1: Color	Relative fitness	B = −0.24	.324
Step 2: Color	Mean # of visits	B = −0.01	.810
Step 3: Mean # of visits	Relative fitness	s = 1.68	**<.001**
Color	Relative fitness	s = −0.21	.518
Flower diameter→visits→fitness			
Step 1: Flower diameter	Relative fitness	s = 0.15	**<.001**
Step 2: Flower diameter	Mean # of visits	s = 0.03	**.008**
Step 3: Mean # of visits	Relative fitness	s = 1.52	**<**.**001**
Flower diameter	Relative fitness	s = 0.12	**<**.**001**

Effect size was measured as B, the difference between light‐colored and dark‐colored tubes; for continuous explanatory variables, s is the slope of regression. In step 3, s for color is the difference between slopes of light‐colored and dark‐colored tubes. Significant terms (*p* < .05) are in bold. Fitness trait was analyzed as relative fitness (mean fitness = 1) and was ln‐transformed for the significance testing. Although mediational analysis contains four steps, the third step is included in the fourth; hence, for simplicity, the third and fourth steps are combined.

## 4 DISCUSSION

Pollinator's attraction to the visual signal of flowers was proposed to drive selection on the size and color of the advertisement (Fægri & van der Pijl, 1979; Harder & Johnson, 2009). Here, we show that while floral advertisement size in *L. pubescens* is under positive pollinator‐mediated selection, flower tube color is not simply the result of differential pollinator visitation rate and its associated increase in fitness. Instead, we found indirect relationships between flower tube color and fitness, through possible differential visitation rates in morning and evening, potentially associated with different behaviors. These possibly compromise directional selection regimes for any single color, suggesting either divergent selection or no selection on the flower tube colors—which may allow maintenance of color dimorphism.

Floral advertisement size in *L. pubescens* is shown here to be under positive directional pollinator‐mediated selection, based on the apparent association of floral diameter with pollinator visitation rate (Figure 3i,j) and plant's fitness (Figure 3g,h), and the dependence of plant's fitness on the number of visits (Figure 3a,b). *Usia bicolor* flies preferred to visit plants of *L. pubescens* with larger flowers, both in the morning and in the evening. This suggests that flower advertisement size helps pollinators to detect both food sources and mating meeting points, either in the morning or in the evening. Numerous studies have shown that larger corolla size results in an increase in pollinator's visitations, and consequentially increased plant fitness (e.g., Campbell, Waser, Price, Lynch, & Mitchell, 1991; Conner & Rush, 1996; Eckhart, 1991; Fenster, Cheely, Dudash, & Reynolds, 2006; Sletvold et al., 2010; Young & Stanton, 1990). Most pollinator‐mediated selection studies assumed that (or tested whether) flower size is associated with the food reward in it; here, however, while food reward was not measured, we show that floral size in *L. pubescens* is also associated with signaling for a mating place, which can be considered as an alternative reward. It has been shown theoretically that providing both food reward and reward by mating rendezvous for the pollinators can increase cross pollination of plants and can select for increased advertisement size (Fishman & Hadany, 2013). Thus, pollinator‐mediated selection on flower size in *L. pubescens* may be the outcome of the combination of these two rewards.

The role of dark flower center in the pollination system of *L. pubescens* was not revealed here. Several studies have examined the role of central dark‐color spot in flowers (Dafni et al., 1990; Eisikowitch, 1980; Johnson & Midgley, 1997; Keasar et al., 2010; Lamborn & Ollerton, 2000) but to date, there is no clear explanation for their adaptive role. In *L. pubescens*, both dark and light flowers had similar fitness, and overall visit frequencies were not different between tube‐color morphs. Relative frequency of *U. bicolor* visits to dark‐center flowers was higher in the morning (as opposed to Johnson & Dafni, 1998) in 2010, but fitness was similar between the two morphs in that year. Both morning and evening visits affected fitness (although in 2010 only morning did; Table 3), suggesting similar efficiency of the pollination services in both times. This can explain the persistence of color dimorphism over years and across populations of *L. pubescens* (Wolfe, 2001). Other possible mechanisms that maintain polymorphism within populations include contrasting selection regimes created by different pollinators (Eckhart, Rushing, Hart, & Hansen, 2006; Jorgensen, Petanidou, & Andersson, 2006; Pellegrino, Caimi, Noce, & Musacchio, 2005; Sahli & Conner, 2011), or contrasting selection regimes executed by the same animal that acts as both mutualist (pollinator) and antagonist (seed predator; Kephart, Reynolds, Rutter, Fenster, & Dudash, 2006; Morris, Vazquez, & Chacoff, 2010). In *L. pubescens*, observed differences between mornings and evenings in color preferences (at least in one year) suggest that the same pollinator may execute different selection regimes on color over the day. Divergent selection by differential behavior of the same pollinator was shown only in a few studies in South Africa (e.g., Ellis & Anderson, 2011; Ellis & Johnson, 2010; de Jager & Ellis, 2012) and requires additional studies in other ecosystems to assess its generality.

An open question remains regarding the effect of flower tube color on the behavior of *U. bicolor*. Johnson and Dafni (1998) found that the dark spot was more attractive to the flies in the evening (mating time) and hypothesized that “the dark center of the flowers of *L. pubescens* may play an important role in the attraction of mate‐seeking flies” (Johnson & Dafni, 1998 p.295). However, in this study, we found that the preference for dark‐centered flowers is higher in the morning, when feeding is the major behavior, suggesting that the dark‐center signals for food reward. Hence, an alternative hypothesis can be that the dark center serves as mimicry for another feeding fly, and not for a mating partner. This supports the hypothesis of dark center in flowers as a signal for food source (Eisikowitch, 1980). In contrast, light‐colored tubes may signal empty flower. We have observed a significant fraction of flies performing “waiting” behavior in the evening. We propose that this is part of the mating strategy, and can be either active search or waiting behavior. Mating flies may look for empty flowers, which are better perceived when the center of the flower is light colored, due to the contrast between the dark color of the fly and the light color of the flower (Figure 1). The flies actively looking for a partner will prefer a flower occupied by a partner, and may land in dark‐centered flowers as well, mistaking the dark spot for a mating partner. Overall, this complex behavior and the associated color‐pattern choice can be the basis for the selection that maintains color dimorphism in *L. pubescens*.

Our results suggest that *U. bicolor* flies act as the selection agents on floral size in *L. pubescens*. Pollinator‐mediated selection on floral color and its role in maintaining color dimorphism, however, were not found here. This may be due to the effect of color pattern on pollinators’ behavior at different times of the day, which may obscure directional selection. To summarize, floral traits in *L. pubescens* are shaped by different, sometimes contrasting, selection regimes, mediated by *U. bicolor* fly pollinators, and their changing diurnal behaviors.

## CONFLICT OF INTEREST

None declared.

## AUTHOR CONTRIBUTIONS

ML, LH, and YS conceived and designed the study; ML performed the field study; ML, UO, and YS analyzed the data; ML, UO, LH, and YS wrote the manuscript.

## DATA ACCESSIBILITY

Data available from the Dryad Digital Repository: https://doi.org/10.5061/dryad.6sv46
